# Template method for fabricating interdigitate p-n heterojunction for organic solar cell

**DOI:** 10.1186/1556-276X-7-469

**Published:** 2012-08-21

**Authors:** Jianchen Hu, Yasuhiro Shirai, Liyuan Han, Yutaka Wakayama

**Affiliations:** 1 International Center for Materials Nanoarchitectonics (WPI-MANA), National Institute for Materials Science, 1-1 Namiki, Tsukuba, 305-0044, Japan; 2 Department of Chemistry and Biochemistry, Faculty of Engineering, Kyushu University, 1-1 Namiki, Tsukuba, 305-0044, Japan; 3 Photovoltaic Materials Unit, National Institute for Materials Science, 1-2-1 Sengen, Tsukuba, 305-0047, Japan

**Keywords:** Anodic aluminum oxide, P3TH pillars, P-n junction, Photovoltaic effect

## Abstract

Anodic aluminum oxide (AAO) templates are used to fabricate arrays of poly(3-hexylthiophene) (P3HT) pillars. This technique makes it possible to control the dimensions of the pillars, namely their diameters, intervals, and heights, on a tens-of-nanometer scale. These features are essential for enhancing carrier processes such as carrier generation, exciton diffusion, and carrier dissociation and transport. An interdigitated p-n junction between P3HT pillars and fullerene (C_60_) exhibits a photovoltaic effect. Although the device properties are still preliminary, the experimental results indicate that an AAO template is an effective tool with which to develop organic solar cells because highly regulated nanostructures can be produced on large areas exceeding 100 mm^2^.

## Background

Bulk heterojunction (BHJ) solar cells [1,2] are superior to single- [3] and double-layer cells [4]. The BHJ structure can be formed simply by mixing a donor and acceptor solution. This straightforward technique is advantageous in terms of increasing the donor/acceptor (D/A) interface, which provides the exciton dissociation sites. Meanwhile, a weak point as regards BHJs is that the pathways of the generated carriers are not ensured because of the random phase separation of the respective materials. To ensure exciton dissociation and carrier collection, continuous percolation pathways are required.

An ideal structure would be an interdigitated interface, where the donor and acceptor phases are separate. The diameter and interspatial distance of the pillars should preferably be comparable to the diffusion length of the excitons, which is of the order of 10 nm. Then, the excitons can diffuse to the D/A interface during their lifetime [5]. Furthermore, the interdigitated structure must be aligned perpendicularly to connect with the electrodes so as to provide direct pathways for efficient charge transportation [6,7]. Meanwhile, the film thicknesses should be around 100 to 200 nm to absorb the incident light and to confine the series resistance [8,9]. For these reasons, the dimensions of the interdigitated structures should be carefully designed to enhance photovoltaic effects. Interdigitated structures have been obtained using different techniques including self-organization and nanoimprinting [10,11]. However, there is still room for further optimization of the dimensions [12].

**Figure 1:**
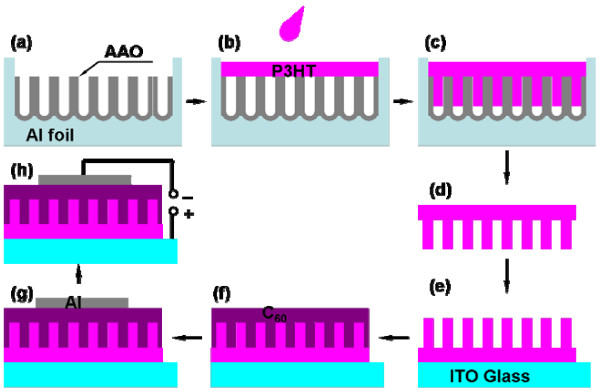
**Polymer solar cell fabrication process.** (**a**) AAO template. (**b**, **c**) Spin coating of P3HT solution and infiltration into pores. (**d**, **e**) Extraction of P3HT pillar film and transfer onto ITO substrate. (**f**, **g**) Vacuum deposition of C_60_ molecules and Al electrode. (**h**) *I*-*V* measurement.

In this study, we employed an anodic aluminum oxide (AAO) template to prepare a poly(3-hexylthiophene) (P3HT)/fullerene (C_60_) interdigitated interface. P3HT absorbs the light from 400 to 700 nm to generate excitons. Meanwhile, C_60_ absorbs the light in the UV range of 300 to 400 nm and acts as an electron acceptor to transport such electrons to the electrode [9,13]. The fabrication process and experimental procedures are described in Figure 1 and the ‘Methods’ section. This strategy has the following merits: Firstly, the pillar size can be well controlled as regards diameter, interval, and height, making it close to the exciton diffusion length. Secondly, a carrier pathway can be formed, which is directly connected to the electrodes. Finally, self-standing pillars can be fabricated uniformly over a large area. Besides, with this technology, costly imprinting equipment and cumbersome processes like dry etching [14,15] are avoidable. By employing these advantages, we demonstrate the fine-tuning of nanostructures for organic solar cells and report preliminary device properties.

## Methods

An AAO template was prepared by a conventional procedure using Al sheet (1 mm in thickness) with three main steps: first anodization, removal of the oxide layer, and second anodization [16]. For the first anodization, a constant voltage of 40 V was applied for 12 h in 0.3 M oxalic acid solution at 0 °C. The alumina pores thus grown were etched away in a mixed solution of phosphoric acid (6% H_3_PO_4_) and chromic acid (1.8% CrO_3_) for 12 h at 60 °C. These procedures were needed to obtain a regular array of alumina dimples. The second anodization was initiated from these dimples and resulted in a highly ordered array of pores. The pore depth can be adjusted with the second anodization, which is performed under the same conditions as the first anodization. A subsequent widening process in 10% (*v*/*v*) phosphoric acid allowed fine-tuning of the pore diameters (Figure 1a).

To prepare the P3HT nanopillars, a P3HT solution (3 wt.% in chlorobenzene) was spin coated on the AAO template at a rotation speed of 3,500 rpm (Figure 1b). The P3HT solution penetrated into the pores through capillary force to form P3HT nanopillars (Figure 1c) [17]. The alumina and Al substrate were removed by immersing the sample in 3 M NaOH solution for 45 min, and as a result, a self-standing P3HT film was obtained with a pillar structure on its surface (Figure 1d). Then, the P3HT film was soaked in a 3 M NaOH solution and rinsed in pure water to remove any remaining alumina particles and impurities.

To fabricate a photovoltaic device, the reverse side of the P3HT pillar film should be attached to an indium tin oxide (ITO) substrate. For this purpose, the surface tension of the ITO substrate was modified by soaking it in chloroform for 40 min [18]. The chloroform treatment increased the affinity of the ITO surface for the P3HT films and made it easier to attach them to the ITO substrates (Figure 1e; see Additional file 1:Figure S1).

C_60_ molecules were deposited on the P3HT pillar surface to form a p-n junction in vacuum with a background pressure of 1 × 10^−6^ Pa (Figure 1f). The deposition rate was 10 to 20 nm/h, which was controlled by the temperature of a Knudsen cell. Then, a 120-nm-thick Al electrode was deposited on the C_60_ film (Figure 1g). The P3HT/C_60_ p-n junction thus prepared was annealed in a vacuum at 180 °C for 20 min. Scanning electron microscope (SEM) images were obtained with an SU8000 Hitachi scanning electron microscope (Minato-ku, Japan). The *I*-*V* curve was measured with a WXS-90S-L2 super solar simulator (WACOM, Fukaya-shi, Japan; Figure 1h). All measurements were performed under AM 1.5 irradiation (100 mW/cm^2^) with a 0.04 cm^2^ active surface area.

## Results and discussion

### Dimensional control of P3HT pillar by AAO template

**Figure 2:**
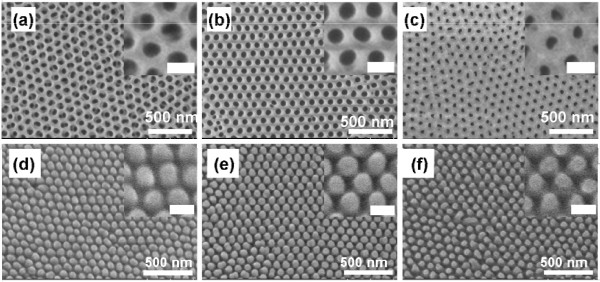
**SEM images of AAO templates and PH3T nanopillars.** (**a**, **b**, **c**) SEM images of AAO templates with different pore diameters of (**a**) 80, (**b**) 60, and (**c)** 40 nm. (**d**, **e**, **f**) SEM images of P3HT nanopillars with diameters of (**d**) 80, (**e**) 60, and (**f**) 40 nm. Samples (**d**, **e**, **f**) were tilted by 30°. The insets show enlarged images (bar length, 100 nm).

Figure 2a,b,c shows SEM images of AAO templates. The diameters of the pores were tuned from 40 to 80 nm by adjusting the widening time from 1 to 10 min. The SEM images in Figure 2d,e,f show P3HT pillars. These images were obtained after removing the AAO templates with NaOH solution. The diameters of the pillars ranged from 40 to 80 nm, and their intervals coincided well with those of the respective templates. In general, the diffusion length of the excitons in organic semiconductors is in the few tens-of-nanometer range. Therefore, the fine-tuning of the diameters of the pillars demonstrated here is advantageous for dimensional optimization in BHJ solar cells.

Importantly, the P3HT pillar heights were very uniform at about 100 nm regardless of diameter. The height should be optimized to maintain mechanical stability and to enhance light absorption. If the pillars are too tall, aggregation and collapse occur (see Additional file 2: Figure S2). Meanwhile, the pillars should be tall enough to promote light absorption. The height of 100 nm was optimized to satisfy these requirements by adjusting the second anodization time to 70 s. Pillar height uniformity is another essential factor as regards device operation. Such highly regulated P3TH pillars were observed over the template area. Consequently, the AAO template was shown to be a powerful technique for controlling the nanoscale dimensions of the P3HT pillars, namely diameter, interval, and height, as well as their uniformity over a wide area of about 100 mm^2^.

### Fabrication of P3HT/C_60_ interdigitated p-n heterojunction and its photovoltaic property

**Figure 3:**
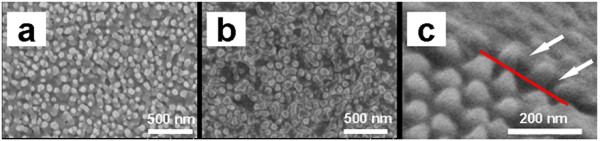
**SEM images of deposited C**_**60**_**films and P3HT/C**_**60**_**heterojunction.** SEM images of (**a**) C_60_ film deposited on P3HT pillars, (**b**) C_60_ film deposited on a flat substrate, and (**c**) cross section of P3HT/C_60_ heterojunction, proving full infiltration of C_60_ between P3HT pillars.

C_60_ molecules were deposited on P3HT pillars in a vacuum to fabricate interdigitated p-n heterojunctions. Figure 3a is a top-view SEM image of the C_60_ film on the P3HT pillars. A SEM image of the C_60_ film deposited on a flat glass substrate is shown in Figure 3b for comparison. Both images show a similar surface morphology; the C_60_ molecules aggregated to form a rough surface, but the entire surface was completely covered with a continuous film. A major concern is the interfacial area between P3HT and C_60_. The C_60_ molecules should be inserted between the P3HT pillars to form an interdigitated interface. To confirm the realization of such an embedded interface structure, a cross-sectional SEM image was obtained as shown in Figure 3c. For this sample, C_60_ molecules were deposited through a shadow mask. The image shows the border between the masked and unmasked areas (red line). The image clearly reveals that the deposited C_60_ molecules infiltrated between the P3HT pillars as indicated by the arrows. After deposition of C_60_ molecules, the thickness of the heterojunction is controlled in a suitable scale (no more than 200 nm; see Additional file 3: Figure S3). The whole thickness consists of three parts: back film thickness, pillar height, and C_60_ film thickness. Thickness of the back film can be controlled by spin coating rate, pillar height can be controlled by AAO template pore depth, and deposition rate and time decide the C_60_ film thickness.

**Figure 4:**
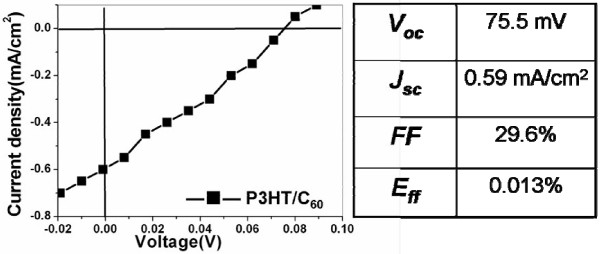
***I*- *V *curve of ITO/P3HT:C**_**60**_**/Al organic photovoltage device.** The properties are shown in the table (nanopillar diameter, 80 nm).

An Al cathode was deposited on the C_60_ film to evaluate the photovoltaic effect. Subsequently, the specimens were thermally annealed to improve the ordering of the polymer chains and crystallinity [19]. Figure 4 shows the *IV* curve of the thus prepared photovoltaic device. The diameter of the P3HT pillars was 80 nm for this measurement. The device properties are summarized in the table in Figure 4. The poor performance is attributed to the sample preparation procedures, e.g., exposure to air during sample handling and transferring the film to ITO in water which may lead to a poor electrical contact. Also, during the removal of AAO template, there might be some impurity splashing to the reverse side of the P3HT pillar film because of the violent reaction, causing a contamination at the interface between ITO and P3HT. Nevertheless, this result demonstrates that the P3HT/C_60_ heterojunction produced by the AAO template can yield a photovoltaic effect.

## Conclusions

We described an AAO template technique for fabricating regular arrays of P3HT pillars and interdigitated p-n junctions of P3HT/C_60_. The feature of this technique is the high controllability of the nanoscale dimensions, such as the diameter, interval, height, and uniformity of the P3HT pillars. That is, these dimensions can be tailored to improve effective light absorption and carrier dissociation and transport. The device properties in this study were preliminary, and there is still room for further improvement. However, the technique we demonstrated here has great potential for use in developing a practical device because nanoscale structures can be fabricated in a large area exceeding 100 mm^2^.

## Abbreviations

AAO: anodic aluminum oxide; BHJ: bulk heterojunction; C_60_: fullerene; D/A: donor/acceptor; ITO: indium tin oxide; P3H: poly(3-hexylthiophene); SEM: scanning electron microscope.

## Competing interests

The authors declare that they have no competing interests.

## Authors’ contributions

JH and YS carried out the experiment, participated in the sequence alignment, and drafted the manuscript. YW and LH participated in the design of this study. All authors read and approved the final manuscript.

## Supplementary Material

Additional file 1 Figure S1.Comparison of ITO substrates before and after modification by chloroform. Before modification, when the ITO substrate approached the P3HT pillar film’s reverse side (a, b), the film was pushed away (c). After modification, when the ITO substrate approached the P3HT pillar film’s reverse side (d, e), the film was attached to the substrate (f). (PDF 1718 kb) Click here for file

Additional file 2 Figure S2.SEM images of P3HT nanowires fabricated by using AAO template prepared by controlling the second anodization time for (a) 2 and (b) 5 min. Click here for file

Additional file 3 Figure S3.SEM image of P3HT/C_60_ heterojunction. Click here for file
