# Supplementary material for: Template method for fabricating interdigitate p-n heterojunction for organic solar cell
Source: Nanoscale Res Lett. 2012 Aug 21;7(1):469. doi: 10.1186/1556-276X-7-469 (PMC3499169; doi:10.1186/1556-276X-7-469)
Supplement: Additional file 1 — Figure S1.Comparison of ITO substrates before and after modification by chloroform. Before modification, when the ITO substrate approached the P3HT pillar film’s reverse side (a, b), the film was pushed away (c). After modification, when the ITO substrate approached the P3HT pillar film’s reverse side (d, e), the film was attached to the substrate (f). (PDF 1718 kb) [file 1556-276X-7-469-S1.pdf]

Chloroform can be used for surface modification for ITO substrate to change the interaction of P3HT pillar film reverse side and ITO substrate.

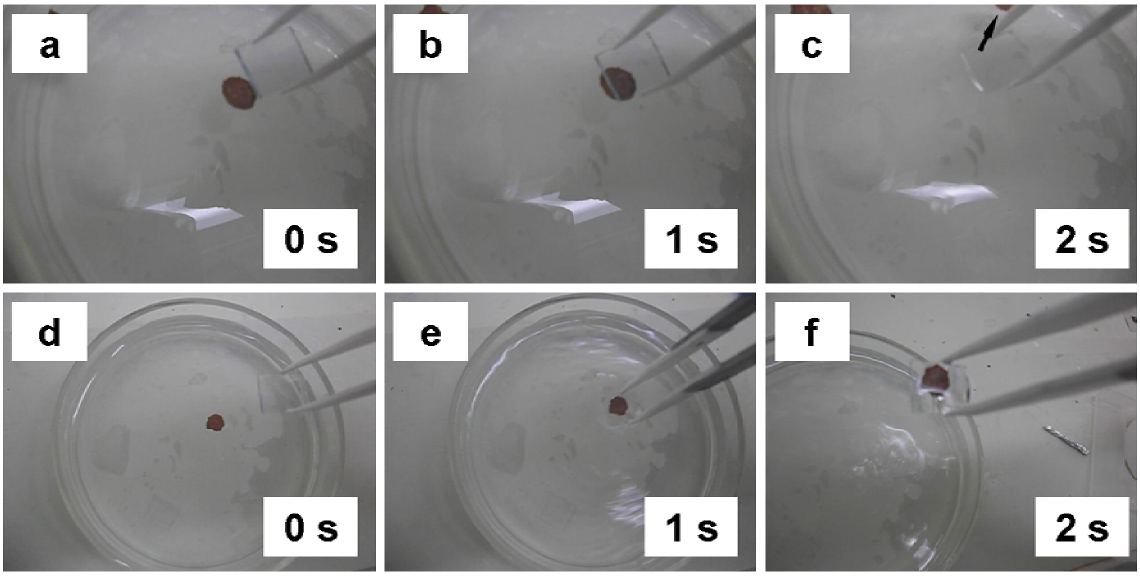

Additional file 1. Comparation of ITO substrates before and after modification by chloroform. Before modification, when ITO substrate approach P3HT pillar film reverse side (a, b), the film was pushed away (c). After modification, when ITO substrate approach P3HT pillar film reverse side (d, e), the film was attached to the substrate (f).
