# Supplementary material for: Template method for fabricating interdigitate p-n heterojunction for organic solar cell
Source: Nanoscale Res Lett. 2012 Aug 21;7(1):469. doi: 10.1186/1556-276X-7-469 (PMC3499169; doi:10.1186/1556-276X-7-469)
Supplement: Additional file 2 — Figure S2.SEM images of P3HT nanowires fabricated by using AAO template prepared by controlling the second anodization time for (a) 2 and (b) 5 min. [file 1556-276X-7-469-S2.pdf]

The 2nd anodization time of AAO template should be controlled to control the depth of pores that decide the length of subsequently formed polymer pillars or nanowires. If the 2nd anodization time is too long, aggregation and collapse of polymer pillars or nanowires happens.

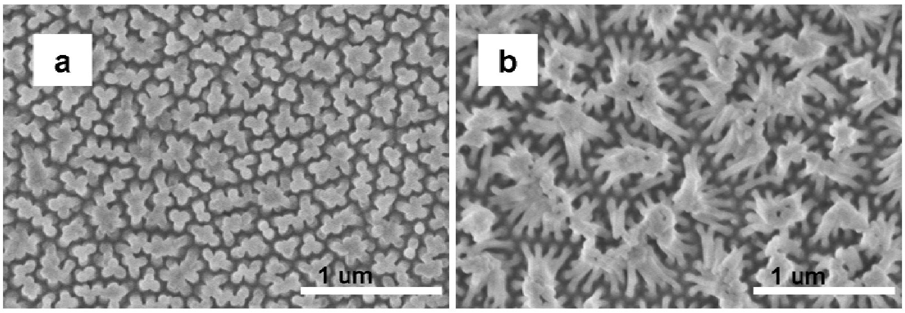

Additional file 2. SEM images of P3HT nanowires fabricated by using AAO template prepared by controlling the 2nd anodization time for a) 2 min and b) 5 min.
