# Supplementary material for: Template method for fabricating interdigitate p-n heterojunction for organic solar cell
Source: Nanoscale Res Lett. 2012 Aug 21;7(1):469. doi: 10.1186/1556-276X-7-469 (PMC3499169; doi:10.1186/1556-276X-7-469)
Supplement: Additional file 3 — Figure S3.SEM image of P3HT/C60 heterojunction. [file 1556-276X-7-469-S3.pdf]

The contrast of P3HT and C_{60} is not so clearly indicated in SEM image of cross section because the the main element for the two materials is same.

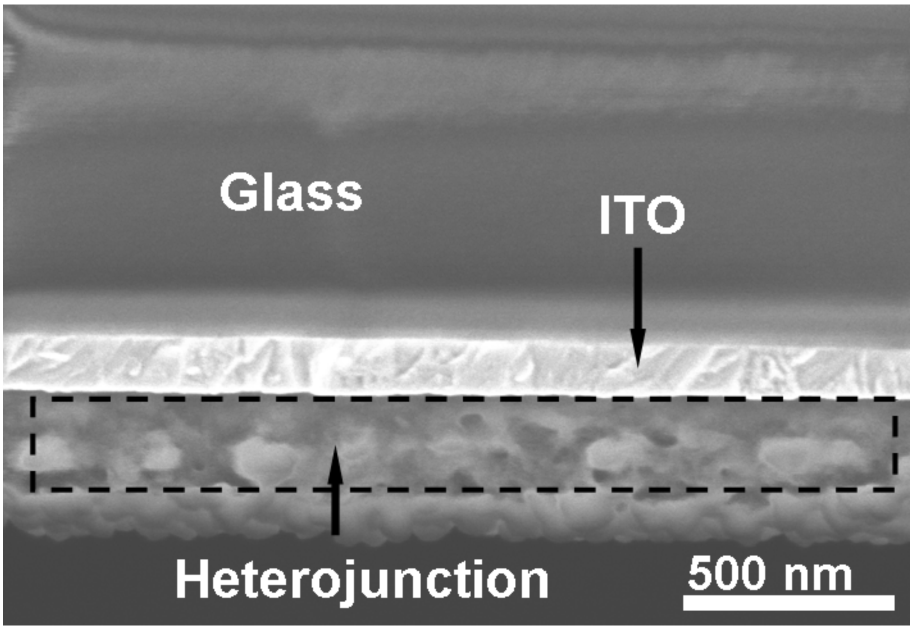

Additional file 3. (Cross section) SEM image of P3HT/ C_{60} heterojunction. It also shows the thickness of heterojunction is less than 200 nm.
